# *tert*-Butoxy­triphenyl­silane

**DOI:** 10.1107/S1600536810002722

**Published:** 2010-01-27

**Authors:** Jonathan O. Bauer, Carsten Strohmann

**Affiliations:** a Anorganische Chemie, Technische Universität Dortmund, Otto-Hahn-Strasse 6, 44227 Dortmund, Germany

## Abstract

The title compound, C_22_H_24_OSi or Ph_3_SiO^*t*^Bu, shows a distorted tetra­hedral coordination sphere around the Si atom. The C—O—Si angle is 135.97 (12)° and the O—Si distance is 1.6244 (13) Å. The mol­ecules are held together by weak inter­actions only. An H⋯H distance of 2.2924 (7) Å is found between aryl H atoms and is the shortest inter­molecular distance in the structure. With regard to the broad applicability of *R*_3_SiO structural motifs in all fields of chemistry, the mol­ecule demonstrates a common model system for silicon centers surrounded by sterically demanding substituents.

## Related literature

For the synthesis of Ph_3_SiO-*t*-Bu, see: Gilman *et al.* (1953 ▶). For the synthesis and structure of Ph_3_SiO-*i*-Pr, see: Wojtczak *et al.* (1996 ▶). For selected transition-metal complexes containing Ph_3_SiO groups, see: Bindl *et al.* (2009 ▶); Johnson *et al.* (2000 ▶); Ruiz *et al.* (2004 ▶); Schweder *et al.* (1999 ▶); Schweder *et al.* (2006 ▶); Wolff von Gudenberg *et al.* (1994 ▶). For selected main-group compounds containing Ph_3_SiO units, see: Apblett & Barron (1993 ▶); Chen *et al.* (2008 ▶); Ferguson *et al.* (1996 ▶, 2005 ▶). For applications of silyl ethers in protecting group chemistry, see: Scheidt *et al.* (2002 ▶); Vintonyak & Maier (2007 ▶). For comparative O—Si distances, see: Bowes *et al.* (2002 ▶); Wojtczak *et al.* (1996 ▶) and for C—Si distances, see: Dilman *et al.* (2004 ▶); Lee *et al.* (2001 ▶); Wojtczak *et al.* (1996 ▶).
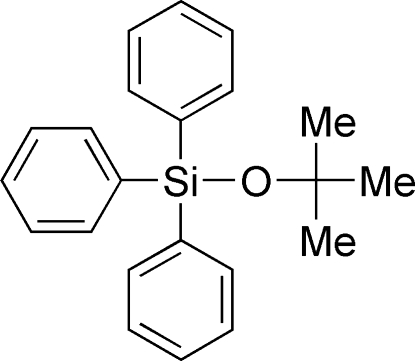


## Experimental

### 

#### Crystal data

C_22_H_24_OSi
*M* *_r_* = 332.5
Monoclinic, 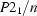

*a* = 9.8054 (12) Å
*b* = 20.201 (7) Å
*c* = 10.231 (2) Å
β = 111.311 (18)°
*V* = 1888.0 (8) Å^3^
*Z* = 4
Mo *K*α radiation
μ = 0.13 mm^−1^
*T* = 173 K
0.30 × 0.20 × 0.20 mm

#### Data collection

Oxford Diffraction Xcalibur S diffractometer
Absorption correction: multi-scan (*CrysAlis RED*; Oxford Diffraction, 2006 ▶) *T*_min_ = 0.962, *T*_max_ = 0.975
23180 measured reflections
4203 independent reflections
2597 reflections with *I* > 2σ(*I*)
*R*_int_ = 0.045

#### Refinement

*R*[*F* ^2^ > 2σ(*F* ^2^)] = 0.042
*wR*(*F* ^2^) = 0.100
*S* = 0.89
4203 reflections
220 parameters
H-atom parameters constrained
Δρ_max_ = 0.37 e Å^−3^
Δρ_min_ = −0.24 e Å^−3^

### 

Data collection: *CrysAlis CCD* (Oxford Diffraction, 2006 ▶); cell refinement: *CrysAlis RED* (Oxford Diffraction, 2006 ▶); data reduction: *CrysAlis RED*; program(s) used to solve structure: *SHELXS97* (Sheldrick, 2008 ▶); program(s) used to refine structure: *SHELXL97* (Sheldrick, 2008 ▶); molecular graphics: *ORTEP-3* (Farrugia, 1997 ▶); software used to prepare material for publication: *SHELXL97*.

## Supplementary Material

Crystal structure: contains datablocks I, global. DOI: 10.1107/S1600536810002722/fb2173sup1.cif

Structure factors: contains datablocks I. DOI: 10.1107/S1600536810002722/fb2173Isup2.hkl

Additional supplementary materials:  crystallographic information; 3D view; checkCIF report

## Figures and Tables

**Fig. 1.**
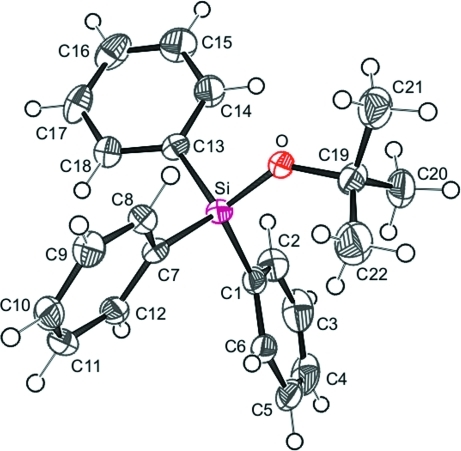
The title molecule tert-butoxytriphenylsilane with the displacement ellipsoids drawn at the 50% probability level.

## supplementary crystallographic information

### Comment

Siloxy groups are versatile structural subunits both in organic and inorganic chemistry, *e.g.* in the design of transition metal based catalysts (Bindl *et al.*, 2009; Schweder *et al.*, 1999) such as in the recently developed molybdenum triphenylsiloxide complex, which serves as a catalyst for alkyne metathesis (Bindl *et al.*, 2009). In the light of the impressive attainment in natural product synthesis, silyl ethers in addition proved to be an essential structural element in their role as commonly used protecting groups as they are applied for alcohol functionalities (Scheidt *et al.*, 2002; Vintonyak & Maier, 2007). Aside from several known *x*-ray structures of triphenylsiloxy substituted transition metal complexes (Bindl *et al.*, 2009; Johnson *et al.*, 2000; Ruiz *et al.*, 2004; Schweder *et al.*, 1999; Schweder *et al.*, 2006; Wolff von Gudenberg *et al.*, 1994), there have also been reported some corresponding main group compounds (Apblett & Barron, 1993; Ferguson *et al.*, 1996; Ferguson *et al.*, 2005; Wojtczak *et al.*, 1996) as well as palladium allyl species that contain triphenylsilyl ether subunits (Chen *et al.*, 2008).

The title compound, *tert*-butoxytriphenylsilane, was originally synthesized by Gilman *et al.* (1953) by refluxing chlorotriphenylsilane in *tert*-butyl alcohol in the presence of dimethylaniline. While the crystal structure of Ph_3_SiO-*i*-Pr was found to be already determined (Wojtczak *et al.*, 1996), no structural data about the bulkier substituted Ph_3_SiO-*t*-Bu have been described yet.

The molecule of the title compound features a distorted tetrahedral coordination around the silicon center. Contrary to the virtually tetrahedral O—Si—C7 and O—Si—C1 bond angles of 111.34 (8)° and 112.63 (8)°, respectively, the O—Si—C13 angle with a value of 102.31 (8)° was found to be significantly smaller. The remarkable sterical hindrance between the bulky *tert*-butoxy substituent and the three phenyl groups is also reflected by the large C19—O—Si bond angle of 135.97 (12)° which is comparable to the C—O—Si angle in the structurally characterized silylenol ether isopropenyloxy[tris(pentafluorophenyl)]silane [138.9 (1)°] (Dilman *et al.*, 2004). However, the value of this angle in both the latter and the title compound is noticeably larger than the respective angle in *iso*-propoxytriphenylsilane [124.8 (1)°] (Wojtczak *et al.*, 1996). In the title structure, the three C—Si bond lengths have values of 1.8545 (19) Å (C7—Si), 1.8541 (18) Å (C13—Si) and 1.8623 (19) Å (C1—Si) and thus are comparable to the distances found in the related systems (Dilman *et al.*, 2004; Lee *et al.*, 2001; Wojtczak *et al.*, 1996). It is also worth mentioning that the interatomic O—Si distance with 1.6244 (13) Å is slightly shorter than those in the reported tetrameric triphenylsilanol [values denoted from 1.6397 (19) Å to 1.646 (2) Å] (Bowes *et al.*, 2002) and the aforementioned Ph_3_SiO-*i*-Pr [1.641 (2) Å] (Wojtczak *et al.*, 1996). The distance between the aryl H4 atomes (H4—H4'; -x+1, -y, -z+2) equals to 2.2924 (7) Å and it was identified as the shortest intermolecular distance in the structure.

### Experimental

Potassium-*tert*-butoxide (503 mg, 4.48 mmol), dissolved in absolute tetrahydrofuran (4 ml), was added dropwise at 0°C to a stirred solution of chlorotriphenylsilane (1.10 g, 3.73 mmol) in 7 ml of absolute tetrahydrofuran. The resulting reaction mixture was stirred for 4 h at room temperature. After saturated aqueous NH_4_Cl solution (10 ml) had been added, the organic layer was separated and the aqueous phase extracted with diethyl ether (2×10 ml and 2×5 ml). The combined ether extracts were washed with water (10 ml) and then dried over anhydrous Na_2_SO_4_. After filtration, all volatiles were removed under reduced pressure to yield 90% (1.11 g) of a white solid. The crude product was purified by Kugelrohr distillation (140°C, 0.8 mbar). Recrystallization of the title compound from diethyl ether resulted in the formation of small and transparent plates in the range of 0.30 × 0.20 × 0.20 mm, suitable for single-crystal *x*-ray studies.

^1^H-NMR (300.1 MHz, CDCl_3_): δ = 1.29 [s, 9H; C(C*H*_3_)_3_], 7.34–7.45 (m, 9H; *H*_aromat._), 7.68–7.71 (m, 6H; *H*_aromat._).

{^1^H}^13^C-NMR (75.5 MHz, CDCl_3_): δ = 32.1 (3 C) [C(*C*H_3_)_3_], 74.1 (1 C) [*C*(CH_3_)_3_], 127.6 (6 C) (*C_meta_*), 129.5 (3 C) (*C_para_*), 135.5 (6 C) (*C_ortho_*), 136.6 (3 C) (*C_ipso_*).

{^1^H}^29^Si-NMR (59.6 MHz, CDCl_3_): δ = -22.2 (1Si).

GC/EI—MS (70 eV): t_R _= 7.06 min; m/*z* (%) = 332 (11) [*M^+^*], 317 (62) [(*M-*Me)^+^], 259 (100) [(Ph_3_Si)^+^], 199 (79) [(Ph_2_SiHO)^+^], 105 (5) [(SiPh)^+^].

Analysis: C_22_H_24_OSi calculated: C 79.47%, H 7.28%; found: C 79.4%, H 7.3%.

### Refinement

All the H atoms could have been discerned in the difference electron density map. However, the H atoms were refined in their idealized geometric positions using the riding model approximation with *U*_iso_(H) = 1.5*U*_eq_(C) for the methyl H atoms and of *U*_iso_(H) = 1.2*U*_eq_(C) for the aryl H atoms. The applied C—H distance constraints: methyl 0.98 Å; aryl 0.95 Å.

### Crystal data

**Table:**

C_22_H_24_OSi	*F*(000) = 712
*M**_r_* = 332.5	*D*_x_ = 1.170 Mg m^−^^3^
Monoclinic, *P*2_1_/*n*	Mo *K*α radiation, λ = 0.71073 Å
Hall symbol: -P 2yn	Cell parameters from 6858 reflections
*a* = 9.8054 (12) Å	θ = 2.2–29.1°
*b* = 20.201 (7) Å	µ = 0.13 mm^−^^1^
*c* = 10.231 (2) Å	*T* = 173 K
β = 111.311 (18)°	Block, colourless
*V* = 1888.0 (8) Å^3^	0.30 × 0.20 × 0.20 mm
*Z* = 4	

### Data collection

**Table:**

Oxford Diffraction Xcalibur S diffractometer	4203 independent reflections
Radiation source: Enhance (Mo) X-ray Source	2597 reflections with *I* > 2σ(*I*)
graphite	*R*_int_ = 0.045
ω scans	θ_max_ = 27.2°, θ_min_ = 2.4°
Absorption correction: multi-scan (*CrysAlis RED*; Oxford Diffraction, 2006)	*h* = −12→12
*T*_min_ = 0.962, *T*_max_ = 0.975	*k* = −26→25
23180 measured reflections	*l* = −13→13

### Refinement

**Table:**

Refinement on *F*^2^	Primary atom site location: structure-invariant direct methods
Least-squares matrix: full	Secondary atom site location: difference Fourier map
*R*[*F*^2^ > 2σ(*F*^2^)] = 0.042	Hydrogen site location: difference Fourier map
*wR*(*F*^2^) = 0.100	H-atom parameters constrained
*S* = 0.89	*w* = 1/[σ^2^(*F*_o_^2^) + (0.0535*P*)^2^] where *P* = (*F*_o_^2^ + 2*F*_c_^2^)/3
4203 reflections	(Δ/σ)_max_ < 0.001
220 parameters	Δρ_max_ = 0.37 e Å^−^^3^
0 restraints	Δρ_min_ = −0.24 e Å^−^^3^

### Special details

**Table:**

Experimental. empirical absorption correction using spherical harmonics, implemented in SCALE3 ABSPACK scaling algorithm
Geometry. All e.s.d.'s (except the e.s.d. in the dihedral angle between two l.s. planes) are estimated using the full covariance matrix. The cell e.s.d.'s are taken into account individually in the estimation of e.s.d.'s in distances, angles and torsion angles; correlations between e.s.d.'s in cell parameters are only used when they are defined by crystal symmetry. An approximate (isotropic) treatment of cell e.s.d.'s is used for estimating e.s.d.'s involving l.s. planes.
Refinement. Refinement of *F*^2^ against ALL reflections. The weighted *R*-factor *wR* and goodness of fit *S* are based on *F*^2^, conventional *R*-factors *R* are based on *F*, with *F* set to zero for negative *F*^2^. The threshold expression of *F*^2^ > σ(*F*^2^) is used only for calculating *R*-factors(gt) *etc*. and is not relevant to the choice of reflections for refinement. *R*-factors based on *F*^2^ are statistically about twice as large as those based on *F*, and *R*-factors based on ALL data will be even larger.

### Fractional atomic coordinates and isotropic or equivalent isotropic displacement parameters (Å^2^)

**Table:**

	*x*	*y*	*z*	*U*_iso_*/*U*_eq_	
C1	0.27284 (19)	0.12279 (9)	0.61661 (17)	0.0278 (4)	
C2	0.1958 (2)	0.08722 (10)	0.68339 (19)	0.0403 (5)	
H2	0.0935	0.0808	0.637	0.048*	
C3	0.2644 (3)	0.06106 (11)	0.8152 (2)	0.0556 (6)	
H3	0.2098	0.0363	0.8583	0.067*	
C4	0.4122 (3)	0.07079 (12)	0.8846 (2)	0.0615 (7)	
H4	0.4593	0.0535	0.9764	0.074*	
C5	0.4911 (3)	0.10533 (11)	0.8215 (2)	0.0506 (6)	
H5	0.5932	0.1119	0.8692	0.061*	
C6	0.4227 (2)	0.13062 (9)	0.68881 (19)	0.0358 (5)	
H6	0.4789	0.154	0.6454	0.043*	
C7	0.27111 (17)	0.23135 (8)	0.41104 (17)	0.0238 (4)	
C8	0.27112 (18)	0.24872 (9)	0.27948 (18)	0.0285 (4)	
H8	0.223	0.2207	0.2017	0.034*	
C9	0.3389 (2)	0.30526 (9)	0.25882 (19)	0.0331 (4)	
H9	0.3368	0.3162	0.1678	0.04*	
C10	0.40953 (19)	0.34582 (9)	0.3708 (2)	0.0349 (4)	
H10	0.4588	0.3843	0.3576	0.042*	
C11	0.40926 (19)	0.33105 (9)	0.50166 (19)	0.0337 (5)	
H11	0.4562	0.3598	0.5784	0.04*	
C12	0.34073 (19)	0.27453 (9)	0.52133 (18)	0.0292 (4)	
H12	0.3409	0.2647	0.6122	0.035*	
C13	−0.01766 (18)	0.17492 (9)	0.40683 (17)	0.0262 (4)	
C14	−0.1271 (2)	0.12846 (10)	0.3473 (2)	0.0400 (5)	
H14	−0.101	0.0856	0.3261	0.048*	
C15	−0.2717 (2)	0.14281 (12)	0.3185 (2)	0.0533 (6)	
H15	−0.3444	0.1101	0.2776	0.064*	
C16	−0.3119 (2)	0.20462 (12)	0.3486 (2)	0.0504 (6)	
H16	−0.4121	0.2147	0.3288	0.061*	
C17	−0.2070 (2)	0.25123 (10)	0.4069 (2)	0.0425 (5)	
H17	−0.2342	0.294	0.4272	0.051*	
C18	−0.0612 (2)	0.23652 (9)	0.43663 (18)	0.0315 (4)	
H18	0.0108	0.2694	0.4784	0.038*	
C19	0.2491 (2)	0.04775 (9)	0.29651 (19)	0.0333 (4)	
C20	0.2387 (2)	−0.01047 (10)	0.3858 (2)	0.0500 (6)	
H20A	0.2889	0.0004	0.4852	0.075*	
H20B	0.285	−0.0493	0.3621	0.075*	
H20C	0.1356	−0.0201	0.3679	0.075*	
C21	0.1796 (3)	0.02943 (11)	0.1418 (2)	0.0527 (6)	
H21A	0.0764	0.018	0.1196	0.079*	
H21B	0.2311	−0.0086	0.1223	0.079*	
H21C	0.1864	0.0671	0.0842	0.079*	
C22	0.4025 (2)	0.06981 (12)	0.3291 (3)	0.0613 (7)	
H22A	0.404	0.1062	0.2664	0.092*	
H22B	0.4607	0.0328	0.3159	0.092*	
H22C	0.4439	0.085	0.4267	0.092*	
O	0.16116 (12)	0.10112 (6)	0.31536 (11)	0.0266 (3)	
Si	0.17626 (5)	0.15575 (2)	0.43643 (5)	0.02347 (13)	

### Atomic displacement parameters (Å^2^)

**Table:**

	*U*^11^	*U*^22^	*U*^33^	*U*^12^	*U*^13^	*U*^23^
C1	0.0329 (10)	0.0245 (10)	0.0250 (9)	0.0035 (8)	0.0092 (8)	−0.0018 (8)
C2	0.0516 (13)	0.0398 (12)	0.0318 (11)	0.0006 (10)	0.0180 (10)	0.0021 (9)
C3	0.094 (2)	0.0419 (14)	0.0394 (13)	0.0094 (13)	0.0348 (14)	0.0088 (11)
C4	0.098 (2)	0.0445 (15)	0.0280 (12)	0.0286 (14)	0.0059 (13)	0.0077 (11)
C5	0.0549 (14)	0.0406 (13)	0.0373 (12)	0.0218 (11)	−0.0060 (11)	−0.0078 (10)
C6	0.0378 (11)	0.0305 (11)	0.0320 (11)	0.0081 (9)	0.0042 (9)	−0.0047 (9)
C7	0.0190 (8)	0.0253 (9)	0.0261 (10)	0.0020 (7)	0.0069 (7)	0.0019 (8)
C8	0.0297 (10)	0.0275 (10)	0.0268 (10)	0.0004 (8)	0.0088 (8)	−0.0013 (8)
C9	0.0383 (11)	0.0327 (11)	0.0314 (11)	0.0013 (9)	0.0162 (9)	0.0046 (9)
C10	0.0326 (10)	0.0257 (10)	0.0460 (12)	−0.0034 (9)	0.0140 (9)	0.0053 (9)
C11	0.0322 (10)	0.0297 (11)	0.0319 (11)	−0.0064 (8)	0.0029 (8)	−0.0030 (8)
C12	0.0299 (10)	0.0293 (10)	0.0250 (9)	−0.0008 (8)	0.0060 (8)	0.0012 (8)
C13	0.0251 (9)	0.0294 (10)	0.0256 (9)	0.0010 (8)	0.0108 (7)	0.0060 (8)
C14	0.0265 (10)	0.0374 (12)	0.0571 (13)	−0.0021 (9)	0.0164 (9)	−0.0025 (10)
C15	0.0244 (10)	0.0573 (16)	0.0770 (16)	−0.0071 (11)	0.0170 (11)	0.0003 (13)
C16	0.0267 (11)	0.0609 (16)	0.0673 (15)	0.0102 (11)	0.0214 (11)	0.0186 (13)
C17	0.0437 (12)	0.0417 (13)	0.0516 (13)	0.0170 (11)	0.0285 (10)	0.0152 (10)
C18	0.0341 (10)	0.0313 (11)	0.0331 (11)	0.0008 (9)	0.0169 (8)	0.0034 (9)
C19	0.0318 (10)	0.0343 (11)	0.0334 (11)	0.0086 (9)	0.0113 (8)	0.0021 (9)
C20	0.0624 (15)	0.0318 (12)	0.0506 (13)	0.0106 (11)	0.0142 (11)	0.0014 (10)
C21	0.0675 (16)	0.0492 (14)	0.0397 (13)	0.0188 (12)	0.0176 (11)	−0.0022 (11)
C22	0.0397 (13)	0.0515 (15)	0.0967 (19)	0.0006 (12)	0.0295 (13)	−0.0100 (13)
O	0.0239 (6)	0.0260 (7)	0.0280 (7)	0.0030 (5)	0.0070 (5)	−0.0026 (5)
Si	0.0205 (2)	0.0252 (3)	0.0237 (3)	−0.0009 (2)	0.00684 (18)	−0.0001 (2)

### Geometric parameters (Å, °)

**Table:**

C1—C2	1.389 (2)	C13—C14	1.388 (3)
C1—C6	1.393 (2)	C13—Si	1.8544 (17)
C1—Si	1.8626 (18)	C14—C15	1.371 (3)
C2—C3	1.375 (3)	C14—H14	0.95
C2—H2	0.95	C15—C16	1.378 (3)
C3—C4	1.376 (3)	C15—H15	0.95
C3—H3	0.95	C16—C17	1.362 (3)
C4—C5	1.366 (3)	C16—H16	0.95
C4—H4	0.95	C17—C18	1.382 (2)
C5—C6	1.375 (3)	C17—H17	0.95
C5—H5	0.95	C18—H18	0.95
C6—H6	0.95	C19—O	1.437 (2)
C7—C8	1.391 (2)	C19—C22	1.485 (3)
C7—C12	1.393 (2)	C19—C20	1.516 (3)
C7—Si	1.8549 (18)	C19—C21	1.524 (3)
C8—C9	1.376 (2)	C20—H20A	0.98
C8—H8	0.95	C20—H20B	0.98
C9—C10	1.374 (2)	C20—H20C	0.98
C9—H9	0.95	C21—H21A	0.98
C10—C11	1.373 (3)	C21—H21B	0.98
C10—H10	0.95	C21—H21C	0.98
C11—C12	1.376 (2)	C22—H22A	0.98
C11—H11	0.95	C22—H22B	0.98
C12—H12	0.95	C22—H22C	0.98
C13—C18	1.385 (2)	O—Si	1.6251 (12)
			
C2—C1—C6	117.08 (17)	C14—C15—H15	120
C2—C1—Si	120.00 (14)	C16—C15—H15	120
C6—C1—Si	122.90 (14)	C17—C16—C15	119.53 (19)
C3—C2—C1	121.4 (2)	C17—C16—H16	120.2
C3—C2—H2	119.3	C15—C16—H16	120.2
C1—C2—H2	119.3	C16—C17—C18	120.27 (19)
C2—C3—C4	119.9 (2)	C16—C17—H17	119.9
C2—C3—H3	120	C18—C17—H17	119.9
C4—C3—H3	120	C17—C18—C13	121.47 (18)
C5—C4—C3	120.0 (2)	C17—C18—H18	119.3
C5—C4—H4	120	C13—C18—H18	119.3
C3—C4—H4	120	O—C19—C22	110.67 (16)
C4—C5—C6	120.0 (2)	O—C19—C20	109.03 (15)
C4—C5—H5	120	C22—C19—C20	112.45 (17)
C6—C5—H5	120	O—C19—C21	104.93 (14)
C5—C6—C1	121.6 (2)	C22—C19—C21	109.93 (17)
C5—C6—H6	119.2	C20—C19—C21	109.56 (17)
C1—C6—H6	119.2	C19—C20—H20A	109.5
C8—C7—C12	116.97 (16)	C19—C20—H20B	109.5
C8—C7—Si	121.30 (13)	H20A—C20—H20B	109.5
C12—C7—Si	121.69 (13)	C19—C20—H20C	109.5
C9—C8—C7	121.87 (17)	H20A—C20—H20C	109.5
C9—C8—H8	119.1	H20B—C20—H20C	109.5
C7—C8—H8	119.1	C19—C21—H21A	109.5
C10—C9—C8	119.50 (17)	C19—C21—H21B	109.5
C10—C9—H9	120.3	H21A—C21—H21B	109.5
C8—C9—H9	120.3	C19—C21—H21C	109.5
C11—C10—C9	120.31 (17)	H21A—C21—H21C	109.5
C11—C10—H10	119.8	H21B—C21—H21C	109.5
C9—C10—H10	119.8	C19—C22—H22A	109.5
C10—C11—C12	119.81 (17)	C19—C22—H22B	109.5
C10—C11—H11	120.1	H22A—C22—H22B	109.5
C12—C11—H11	120.1	C19—C22—H22C	109.5
C11—C12—C7	121.52 (17)	H22A—C22—H22C	109.5
C11—C12—H12	119.2	H22B—C22—H22C	109.5
C7—C12—H12	119.2	C19—O—Si	135.97 (11)
C18—C13—C14	116.93 (16)	O—Si—C13	102.32 (7)
C18—C13—Si	122.11 (13)	O—Si—C7	111.33 (7)
C14—C13—Si	120.89 (14)	C13—Si—C7	109.98 (8)
C15—C14—C13	121.72 (19)	O—Si—C1	112.61 (7)
C15—C14—H14	119.1	C13—Si—C1	111.02 (8)
C13—C14—H14	119.1	C7—Si—C1	109.42 (8)
C14—C15—C16	120.1 (2)		
